# Piperacillin Steady State Concentrations in Target Tissues Relevant for PJI Treatment—A Randomized Porcine Microdialysis Study Comparing Continuous Infusion with Intermittent Short-Term Infusion

**DOI:** 10.3390/antibiotics12030577

**Published:** 2023-03-14

**Authors:** Hans Christian Rasmussen, Pelle Hanberg, Martin Knudsen, Sara Kousgaard Tøstesen, Andrea René Jørgensen, Elisabeth Krogsgaard Petersen, Kristina Öbrink-Hansen, Kjeld Søballe, Maiken Stilling, Mats Bue

**Affiliations:** 1 Department of Clinical Medicine, Aarhus University, 8200 Aarhus N, Denmark; pellehanberg@clin.au.dk (P.H.); martin.b.knudsen@hotmail.com (M.K.); sartoe@clin.au.dk (S.K.T.); anjo@clin.au.dk (A.R.J.); elisabethkp@clin.au.dk (E.K.P.); kristina.obrink.hansen@auh.rm.dk (K.Ö.-H.); kjeld@soballe.dk (K.S.); maiken.stilling@clin.au.dk (M.S.); matsbue@clin.au.dk (M.B.); 2 Aarhus Denmark Microdialysis Research (ADMIRE), Orthopaedic Research Laboratory, Aarhus University Hospital, 8200 Aarhus N, Denmark; 3 Department of Infectious Diseases, Internal Medicine, Gødstrup Hospital, 7400 Herning, Denmark; 4 Department of Orthopaedic Surgery, Aarhus University Hospital, 8200 Aarhus N, Denmark

**Keywords:** pharmacokinetics, piperacillin, prosthetic joint infections, microdialysis, *Pseudomonas aeruginosa*, porcine model

## Abstract

(1) Introduction: Piperacillin is a common antibiotic choice in the treatment of periprosthetic joint infections (PJI) caused by *Pseudomonas aeruginosa*. The aim of this study was to assess and compare the time with free piperacillin concentration above the minimum inhibitory concentration (*f*T > MIC) at steady state in target tissues relevant for PJI treatment following continuous and intermittent short-term infusion. (2) Methods: 16 pigs were randomized to receive either continuous or intermittent short-term infusion of piperacillin. Steady state piperacillin concentrations were assessed using microdialysis in tibial cortical bone, tibial cancellous bone, synovial fluid of the knee joint, and subcutaneous tissue. MIC-targets of 4, 8, 16, and 64 mg/L were applied. Plasma samples were obtained as reference. (3) Results: Continuous infusion resulted in longer *f*T > MIC for MIC targets of 4 mg/L and 8 mg/L compared to intermittent short-term infusion in all compartments with the exception of tibial cortical bone. For the MIC-target of 16 mg/L, continuous infusion resulted in a longer *f*T > MIC in all compartments except for the bone compartments. No differences between groups were seen when applying a MIC-target of 64 mg/L. (4) Conclusions: An aggressive dosing strategy may be necessary to obtain sufficient piperacillin concentrations in all bone compartments, particularly if more aggressive targets are applied. Based on the present study, continuous infusion should be considered in the treatment of PJI.

## 1. Introduction

Periprosthetic joint infection (PJI) is a devastating complication to arthroplasty surgery with a reported incidence of 1–2% following primary surgery [1]. Of these, approximately 1–3% are caused by the gram-negative bacteria *Pseudomonas aeruginosa* (*P. aeruginosa*) [2,3,4]. *P. aeruginosa* is a particularly challenging pathogen to treat due to emerging multi-drug resistant strains [5]. Essential for current PJI treatment protocols are: (1) surgery—either one-stage exchange, two-stage exchange, or a combination of debridement, antibiotics, irrigation, and retention (DAIR) [6]. Preferences vary in relation to PJI presentation and geographic location; and (2) sufficient perioperative systemic antibiotic regiments [7]. The definition of a sufficient antibiotic regimen is the ability to reach and maintain an adequate pharmacokinetic profile in relevant target tissues. 

The broad-spectrum beta-lactam antibiotic, piperacillin, often administered in combination with tazobactam, is a key drug in the treatment of *P. aeruginosa* infections [8]. Piperacillin is a time-dependent antibiotic, meaning that the effect is best correlated to the time the free drug concentration is above the minimal inhibitory concentration at target sites (*f*T > MIC) [9]. To sample and evaluate target site *f*T > MIC of piperacillin, microdialysis is an established method for sampling of target site antibiotic concentrations. Microdialysis is particularly advantaged by delivering high temporal resolution concentration-time profiles of the free drug concentration simultaneously from multiple target sites [10,11]. 

Intermittent short-term infusion of piperacillin/tazobactam (pip/tazo) continues to be the standard clinical practice in most orthopedic settings when indicated for PJI treatment. Nevertheless, continuous infusion has been shown to be superior in terms of longer *f*T > MIC, reduced mortality, and increased cure rates in critically ill patients [12,13,14,15,16]. Evaluation of piperacillin concentrations in target tissues relevant for PJI treatment following continuous infusion is lacking and may be included in future dosing regiments for piperacillin [17].

This porcine study aimed to employ microdialysis for assessment and comparison of piperacillin steady state *f*T > MIC in target tissues relevant for PJI treatment: tibial cortical bone, tibial cancellous bone, synovial fluid of the knee joint, subcutaneous tissue, and plasma following continuous and intermittent short-term infusion.

## 2. Results

**Figure 1:**
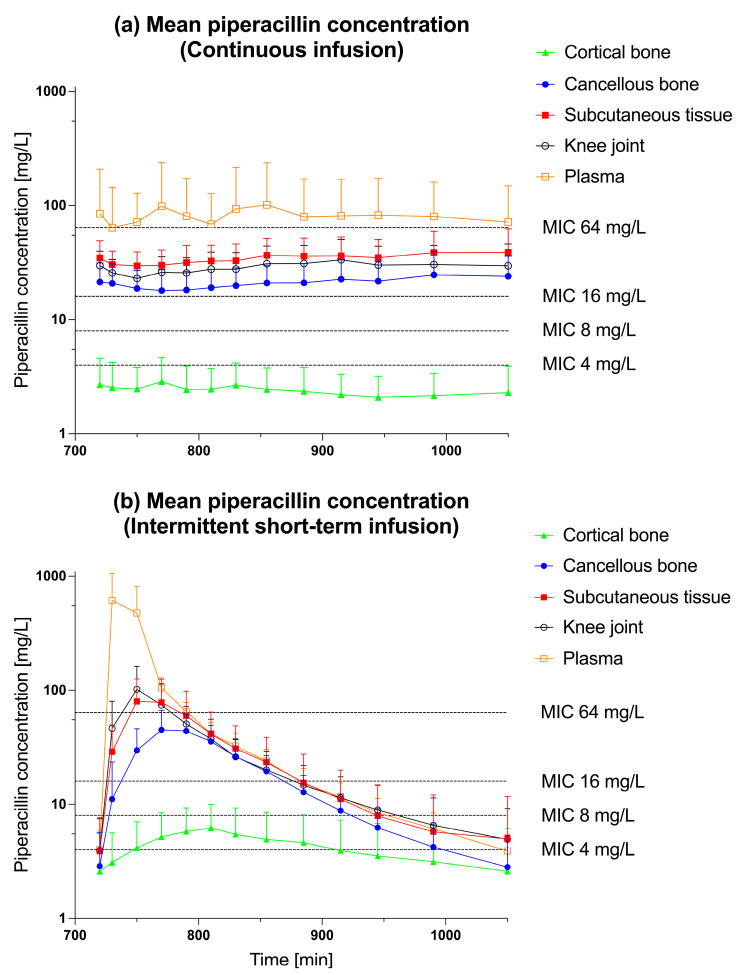
Mean piperacillin concentration–time profiles on log scale following continuous infusion (**a**) and intermittent short-term infusion (**b**). MIC = 4, 8, 16, and 64 mg/L values are inserted for target illustration by dotted lines. Error bars represent 95% CI; upper limit is shown.

All 16 pigs completed the study. Three catheters in subcutaneous tissue and two in synovial fluid of the knee joint were excluded due to malfunctioning membranes. One tibial cortical bone catheter and one cancellous bone catheter were excluded due to incorrect drill hole placement, as assessed on post-mortem CT scans, hence 57 of 64 catheters were used for analysis. Relative recovery (RR) from 14 out of 57 catheters could not be determined reliably, as they were negative or <20% [18]. However, the dialysate piperacillin concentration resembled the concentrations of the remaining catheters in the given compartment. Hence, the group compartment mean RR was applied. The mean RR (SD) across groups for each compartment were 0.55 (0.28) for tibial cortical bone, 0.56 (0.22) for tibial cancellous bone, 0.46 (0.16) for subcutaneous tissue, and 0.53 (0.20) for the synovial fluid of the knee joint. Mean piperacillin concentration–time profiles for the third dosing interval are shown in Figure 1.

The mean *f*T > MIC for the third dosing interval is summarized in Table 1 for both administration groups and all compartments. Table 1 presents mean *f*T > MIC values, calculated separately for each animal and each compartment, whereas Figure 1 depicts the mean piperacillin concentrations in each compartment. For MIC = 4 mg/L, across all compartments, except for tibial cortical bone, continuous infusion resulted in a *f*T > MIC of 100% compared to 74–91% for the intermittent short-term infusion group. For MIC = 8 mg/L, continuous infusion resulted in longer *f*T > MIC across all compartments, except for tibial cortical bone. For MIC = 16 mg/L, continuous infusion resulted in longer *f*T > MIC for all compartments except the two bone compartments. Tibial cortical bone showed no differences in *f*T > MIC for MIC = 4, 8, and 16 mg/L between the two administration groups. When applying MIC = 64 mg/L, no differences in *f*T > MIC were seen between the two administration groups. 

The key pharmacokinetic parameters: area under the curve (AUC_12–18 h_), peak drug concentration (C_max_), tissue penetration (AUC_tissue_/AUC_plasma_), and time until peak drug concentration (T_max_) are presented in Table 2. Continuous infusion resulted in a higher median AUC_12–18 h_ for synovial fluid of the knee joint and subcutaneous tissue compared to intermittent short-term infusion. With the exception of tibial cortical bone, median C_max_ was lower for the continuous infusion group compared to the intermittent short-term infusion group for all compartments. The median tissue penetration was similar for the two administration forms.

## 3. Discussion

Steady state *f*T > MIC and key pharmacokinetic parameters for piperacillin were investigated after continuous and intermittent short-term infusion in target tissues relevant for PJI treatment in a porcine model. Longer *f*T > MIC was observed for MICs of 4, 8, and 16 mg/L, following continuous infusion compared to intermittent short-term infusion in tibial cancellous bone (except for 16 mg/L), synovial fluid of the knee joint, subcutaneous tissue, and plasma. Only tibial cortical bone did not display a difference in *f*T > MIC (4, 8 and 16 mg/L) between the two administration groups. Applying the aggressive target of 64 mg/L (4 × MIC) did not result in any differences in observed *f*T > MIC between the administration forms.

Although appropriate and sufficient antibiotic treatment is considered paramount in PJI treatment, no definitive correlation between antibiotic target site exposure and treatment success exist, as clinical data are lacking [17,19]. However, infected tissue has an impaired and altered blood supply. In bone infections, this is believed to origin from suppurative inflammation with local tissue destruction, osseous sequestration, and increased intraosseous pressure [20]. Previous studies have shown impaired cefuroxime and vancomycin penetration at the site of infection in implant associated *Staphylococcus aureus* infections in a porcine model [21,22]. As the present results are obtained in healthy tissues, they may not readily be translated to an infected PJI setting, where a lower penetration may be expected. Hence, the relationship between piperacillin concentrations at the site of infection and clinical outcome is a needed target for future studies.

A definitive consensus about the optimal *fT >* MIC for piperacillin in PJI settings does not exist [9]. The European Committee on Antimicrobial Susceptibility (EUCAST) reports a required *f*T > MIC for piperacillin of 30−35% to achieve bacteriostasis based mainly on experimental and in vitro data, while clinical data suggest that 100% *f*T > MIC, or even 100% *f*T > 4 × MIC, is needed in critically ill patients to achieve maximal bactericidal effect [23,24,25]. Aggressive targets may be warranted in critically ill patients to suppress resistant subpopulations and overcome decreased susceptibility resulting from prior antibiotic treatments [19,25]. However, the feasibility and relevance of implementing aggressive targets in PJI settings, such as 4 × MIC, is currently lacking sufficient data [25].

The data from the present study suggest that continuous infusion is superior in achieving longer *f*T > MIC compared to intermittent short-term infusion. However, in order to reach a target of 100% *f*T > MIC (MIC: 16 mg/L) in all the bone compartments or 100% 4 × MIC in any compartment, a more aggressive dosing strategy may be necessary. Our results and previous studies demonstrate that continuous piperacillin infusion improves therapeutic exposure and prolongs *f*T > MIC compared to intermittent short-term infusion [14,15,16,26]. Albeit largely reliant on target MIC, piperacillin continuous infusion holds a potential to maximize target achievement whilst minimizing total daily dose [15]. Furthermore, continuous infusion is easy and inexpensive, and potentially harmful concentrations of piperacillin (Neurotoxicity has been associated with plasma concentrations of 157 mg/L, following continuous infusion, and concentrations above 361 mg/L, following intermittent short-term infusion.) could be mitigated by applying continuous infusion [27,28]. In the present study, the mean plasma concentration for the continuous infusion group remained below both proposed neurotoxicity thresholds throughout the entire study period. Yet, it should be noted that staying below the threshold would require evaluation of plasma concentrations, especially in patients with impaired kidney function, which is not standard practice in many orthopedic wards [29]. 

Another commonly used dosing regimen of piperacillin is prolonged/extended infusion often defined as a minimum infusion time of three to four hours [26]. As shown in subcutaneous tissue with pharmacokinetic modelling, this form of administration likely holds a potential to improve *f*T > MIC [16,30]. Some evidence indicates that prolonged infusion is associated with higher clinical cure rate and lower mortality compared to intermittent short-term infusion. However, the immediate clinical advantages remain largely unclear [26,30].

A recently published study, using the same pig population as in the present study, investigated piperacillin concentrations in compartments relevant for spondylodiscitis treatment [11]. The pharmacokinetic results were similar to the present study, e.g., subcutaneous tissue of the neck showed similar *f*T > MIC compared to the present findings in subcutaneous tissue on the leg [11]. However, for the MIC-target of 16 mg/L, a lower *f*T > MIC was observed in vertebral cancellous bone following both administration forms compared to tibial cancellous bone. This difference may be explained by differences in bone microstructure and blood perfusion, which exemplifies the need for assessment of treatment dependent target tissue concentrations at all relevant anatomical locations.

Previous studies have reported cortical bone/plasma concentration ratios of 0.18–0.2 based on human tissue specimens from uninfected femoral cortical bone (1–1.5 h after pip/tazo bolus) [31,32]. However, these studies utilized tissue specimen homogenization, where the derived concentrations represent total tissue concentrations and not the free and unbound interstitial fraction as obtained with the microdialysis technique. Despite using a different methodology, these results are in line with the observed tibial cortical bone/plasma ratios for the intermittent short-term infusion (bolus) group at comparable static timepoints. However, the observed dynamic median AUC_cortical_/AUC_plasma_ ratios were lower: 0.04 (95% CI: 0.02–0.08) for continuous infusion and 0.07 (95% CI: 0.03–0.14) for intermittent short-term infusion.

A central limitation of the present study is that a higher total piperacillin dose was administered to the continuous infusion group compared to the intermittent short-term group (16 g vs. 12 g), possibly explaining some of the observed pharmacokinetic differences. However, the different dosages were opted for to mirror standard clinical dosing during the two administration forms. Pigs have a similar mineral bone composition to humans, and the bone size allows for microdialysis catheter placement [33]. Yet, the clinical translation of the results is still limited by the fact that this is an experimental study on pigs, approximately five months old and still growing with an active epiphyseal plate, in contrast to the typical adult PJI patient. Furthermore, the pigs were kept in general anesthesia for 18 h, which may have influenced the pharmacokinetic profile of piperacillin. The microdialysis technique displays inherent limitations, including the mandatory correction for RR, which may amplify the pre-analytical and analytical variations of the estimated concentrations.

## 4. Materials and Methods

The study was conducted at the Department of Clinical Medicine, Aarhus University Hospital, and it was carried out in agreement with national and international law and with approval by The Danish Animal Experiments Inspectorate (license No. 2017/15-0201-01184). Chemical analysis was performed at the Department of Clinical Biochemistry, Aarhus University Hospital. To comply with the ARRIVE-guidelines and the 3Rs, data obtained from the present study originated from the same pigs used in a recently published article with a different purpose and target tissues [11]. 

### 4.1. Microdialysis

The microdialysis setup consists of a precision pump containing a perfusion fluid, a microdialysis catheter with a semipermeable membrane, and a collection vial. The microdialysis technique allows for continuous and simultaneous sampling of unbound molecules in the interstitial fluid of virtually any tissue of interest [34,35]. Microdialysis is based on passive diffusion of molecules along the concentration gradient across a semipermeable membrane. The impact of convective forces across the membrane is assumed to be negligible, thus making microdialysis a diffusion-limited process [36]. The catheter is continuously perfused by a perfusion fluid, wherefore an equilibrium across the membrane never occurs. The concentration measured in the collected dialysate therefore only represents a fraction of the total tissue concentration. This fraction is referred to as the relative recovery (RR). The absolute extracellular fluid concentration of the analyte can be calculated as dialysate concentration divided by RR (C_tissue_ = C_dialysate/_RR). Microdialysis equipment was acquired from M Dialysis AB, Stockholm, Sweden. Type 63 Microdialysis Catheters with a molecular cut-off of 20.000 dalton and 107 Microdialysis Pumps were used.

### 4.2. Calibration

Individual calibration of the microdialysis catheters can be performed in a variety of ways [35]. In this study, the internal standard calibration method was opted for, as it allows for continuous assessment of RR throughout the entire sampling period. Benzylpenicillin was chosen as an internal standard based on previous thorough in vitro and in vivo investigations [10]. The microdialysis catheters were perfused with 0.9% saline solution containing 5 mg/L benzylpenicillin for at least 30 min to equilibrate before piperacillin administration [18]. Pump perfusion flowrate was 2 µL/min.

### 4.3. MIC Values

According to EUCAST, the epidemiological cutoff (ECOFF) value for pip/tazo against P. aeruginosa is 16 mg/L. However, the majority of isolates are susceptible to lower concentrations of 8 mg/L and 4 mg/L [37]. On top of this, an aggressive target of 4 × MIC has been proposed for piperacillin in the treatment of critically ill patients [23,25]. Hence, these four targets were applied to evaluate fT > MIC. The ECOFF represents a cut-off value separating susceptible wild-type microorganisms from microorganisms with acquired resistance.

### 4.4. Randomization and Dosing

**Figure 2:**
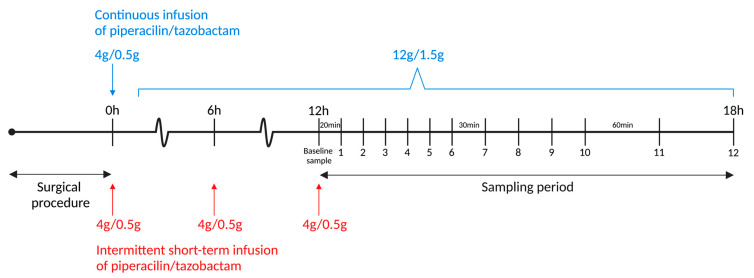
Timeline illustrating piperacillin/tazobactam dosing and sampling in the two administration groups. Created with BioRender.com.

16 female pigs (Danish Landrace, 86–90 kg) were block randomized in pairs of two to receive either continuous or intermittent short-term intravenous infusion of pip/tazo (Fresenius Kabi, Bad Homburg, Germany). In the continuous infusion group, a bolus of 4 g/0.5 g pip/tazo was initially administered at T = 0 over 30 min and followed by a continuous infusion of 12 g/1.5 g during the following 17.5 h (Figure 2). The intermittent short-term infusion group received a bolus of 4 g/0.5 g pip/tazo over 30 min at T = 0 h, T = 6 h and T = 12 h. The total administered piperacillin/tazobactam dose was 16 g/2 g for the continuous infusion group and 12 g/1.5 g for the intermittent short-term infusion group. The dosing regimens were based on recommendations from EUCAST and the Clinical and Laboratory Standards Institute (CLSI), recommending pip/tazo 4 g/0.5 g three times daily for most infections and four times daily in severe cases [8].

### 4.5. Sampling of Steady State Concentrations

Piperacillin plasma half-life in pigs is approximately 73 min (95%CI: 58–90), consequently theoretical steady state would be expected within 5 h to 7.5 h (5 × t_1/2_) [10]. Therefore, steady state was assumed in the third dosing interval, and samples were collected from T = 12 h (720 min) to T = 18 h (1080 min). A sample representing baseline was collected at T = 12 h. Dialysates were collected every 20 min from T = 12 h to T = 14 h, every 30 min from T = 14 h to T = 16 h, and every 60 min from T = 16 h to T = 18 h. Blood samples were drawn from a central venous catheter at the midpoint of each sampling interval. Blood samples were kept at 5 °C for a maximum of 2 h and centrifugated at 3000× *g* for 10 min at 5 °C. Plasma aliquots and dialysates were stored at −80 °C until further analysis. Tazobactam concentrations were not evaluated, since previous studies have indicated similar pharmacokinetics of piperacillin when administered alone and together with tazobactam [38].

### 4.6. Target Tissues, Surgical Procedure, and Anaesthesia 

Tissues relevant for PJI treatment were chosen as targets: tibial cortical bone, tibial cancellous bone, subcutaneous tissue, and synovial fluid of the knee joint. With the pigs in a supine position, the tibial bone of the right hind leg was exposed by an anteromedial incision. A drill hole (∅: 2 mm, depth: 15 mm) was made in the cortical bone at the anterior tibial margin and a 10 mm microdialysis catheter was inserted. Another drill hole (∅: 2 mm, depth: 15 mm) was made in the cancellous bone at the level of the tibial condyles approximately 10 mm distally to the epiphysial line and a 30 mm microdialysis catheter was inserted. Using splitable introducers, a 30 mm microdialysis catheter was placed in the synovial fluid of the knee joint, and a 30 mm microdialysis catheter was placed in the adjacent subcutaneous tissue. Correct catheter placement in the tibial bone was confirmed using intraoperative fluoroscopy, and correct placement of bone drill hole placement was confirmed with post-mortem CT. 

All pigs were kept under general anaesthesia during surgery and the following 18 h, using continuous infusion of fentanyl (0.6–0.75 mg/h, Fresenius Kabi, Germany) and propofol (400–500 mg/h, B. Braun, Melsungen, Germany). Arterial pH was monitored with arterial gas samples and kept in the range of 7.38–7.56 by regulating ventilation. Core body temperature was regulated with cooling and blankets and kept within the range of 35.3–38.8 °C, measured with a rectal thermometer.

### 4.7. Quantification of Piperacillin Concentrations

The free piperacillin and benzylpenicillin concentrations in dialysates, and the free piperacillin concentrations in plasma, were quantified using ultra-high performance liquid chromatography (UHPLC) with UV detection (Agilent Series 1290 Infinity system with a diode array detector, Agilent Technologies, Santa Clara, CA, USA) [10]. The lower limit of quantification was 0.1 mg/L (CV% = 18%) for piperacillin and 0.1 mg/L (CV% = 11%) for benzylpenicillin. The total imprecisions (CV%) for piperacillin (dialysate and plasma) and benzylpenicillin (dialysate) were ≤9%. Linearity was found up to 1000 mg/L for piperacillin and up to 25 mg/L for benzylpenicillin.

### 4.8. Data Analysis

Using linear interpolation, the piperacillin *f*T > MIC was determined for concentrations of 4, 8, 16, and 64 mg/L for each compartment and each animal separately (Microsoft Excel v.2207 build 16.0, Redmond, Washington, USA. Quantile–quantile plots of *f*T > MIC showed a noticeable “ceiling effect” owing to the effect of continuous infusion, as a significant number of observations were equal to the true max of *f*T > MIC = 330 min. Pairwise comparisons of *f*T > MIC were therefore performed using the Wilcoxon rank sum test due to lack of a normal data distribution. For interpretational reasons and comparison with the literature, *f*T > MIC was summarized in minutes and percentages of total sampling time using mean with 95% confidence interval (95%CI).

AUC_12–18 h_, C_max_, AUC_tissue_/AUC_plasma_, and T_max_ were determined by non-compartmental analysis using linear mixed models with repeated-measures analysis of variance (ANOVA) in Stata (v. 17, Statacorp, College Station, TX, USA). The Kenward-Roger’s approximation was used to estimate the degrees of freedom due to small sample size. AUC_12–18 h_ was estimated using the linear up-log down trapezoidal method. Logarithmic transformation was performed on the pharmacokinetic data to attain a normal data distribution. ANOVA analysis was performed assuming equal intergroup and different intercompartmental variance. The data were back-transformed to original scale and summarized as medians with 95%CI. Homogeneity of variance was evaluated with residual vs. fits plots. Normality of residuals was assessed with quantile–quantile plots. Pairwise comparisons were made using linear combination of estimates.

## 5. Conclusions

Piperacillin continuous infusion resulted in longer steady state *f*T > MIC compared to intermittent short-term infusion in tibial cancellous bone (except for 16 mg/L), synovial fluid of the knee joint, subcutaneous tissue, and plasma, but not in the tibial cortical bone when applying MIC-targets of 4, 8 and 16 mg/L. To obtain sufficient piperacillin concentrations in all bone compartments, more aggressive dosing strategies may be necessary, particularly if more aggressive targets are applied. Based on the current findings, and when indicated, continuous infusion of piperacillin should be considered in the treatment of PJI.

## Figures and Tables

**Table 1:** Mean time the free piperacillin concentration was above the minimum inhibitory concentration (*f*T > MIC) in the third dosing interval. T_total_ = 330 min.

	Continuous Infusion			Intermittent Short-Term Infusion			
	[Min] (95%CI)		[%]	[Min] (95%CI)		[%]	*p*-Value
**MIC = 4 mg/L**							
Cortical bone	65	(−37–166)	20	133	(1–266)	040	<0.270
Cancellous bone	330	(330–330)	100	244	(182–306)	074	<0.003
Subcutaneous tissue	330	(330–330)	100	253	(201–305)	077	<0.033
Knee joint	330	(330–330)	100	272	(239–304)	082	<0.019
Plasma	330	(330–330)	100	300	(273–328)	091	<0.001
**MIC = 8 mg/L**							
Cortical bone	0	(0–0)	0	55	(−16–126)	017	<0.154
Cancellous bone	290	(206–374)	88	187	(128–246)	057	<0.019
Subcutaneous tissue	330	(330–330)	100	212	(137–285)	064	<0.008
Knee joint	307	(247–367)	93	220	(160–279)	067	<0.021
Plasma	330	(330–330)	100	238	(200–277)	072	<0.001
**MIC = 16 mg/L**							
Cortical bone	0	(0–0)	000	0	(0–0)	0000	<1.000
Cancellous bone	217	(73–361)	66	119	(78–159)	036	<0.176
Subcutaneous tissue	322	(302–342)	98	152	(82–223)	046	<0.002
Knee joint	275	(134–416)	83	162	(102–222)	049	<0.036
Plasma	322	(302–342)	98	163	(139–187)	049	<0.001
**MIC = 64 mg/L**							
Cortical bone	0	(0–0)	0	0	(0–0)	00	1.000
Cancellous bone	0	(0–0)	0	7	(−6–21)	02	0.467
Subcutaneous tissue	10	(−15–34)	3	30	(−10–69)	09	0.392
Knee joint	0	(0–0)	0	30	(1–60)	09	0.056
Plasma	69	(−38–175)	21	69	(58–79)	021	0.099

**Table 2:** Key pharmacokinetic parameters of piperacillin in the third dosing interval.

	Continuous Infusion		Intermittent Short-Term Infusion		*p*-Value
**Median AUC_12–18 h_, [min**·**mg/mL], (95%CI)**					
Cortical bone	1394	(786–2473)	1649	(896–3036)	0.684
Cancellous bone	12,709	(7870–2,0523)	8009	(5137–12,485)	0.151
Subcutaneous tissue	23,230	(15,557–34,686)	8612	(5946–12,485)	0.002
Knee joint	17,785	(11,327–27,925)	9094	(6049–13,670)	0.033
Plasma	33,573	(19,006–59,303)	24,080	(13,571–42,725)	0.394
**Median C_max_, [m** **g/L], (95%CI)**					
Cortical bone	3	(2–5)	5	(3–9)	0.117
Cancellous bone	22	(14–35)	46	(30–70)	0.026
Subcutaneous tissue	40	(26–63)	77	(51–117)	0.041
Knee joint	35	(20–60)	88	(55–141)	0.016
Plasma	72	(35–145)	499	(247–1011)	0.001
**Median AUC_tissue_/AUC_plasma_,** **(95%CI)**					
Cortical bone	0.04	(0.02–0.08)	0.07	(0.03–0.14)	0.350
Cancellous bone	0.41	(0.21–0.78)	0.33	(0.18–0.62)	0.628
Subcutaneous tissue	0.56	(0.28–1.14)	0.34	(0.17–0.66)	0.280
Knee joint	0.45	(0.22–0.92)	0.38	(0.19–0.76)	0.724
**Median T_max_** **, [** min], (95%CI) *					
Cortical bone	-		809	(750–871)	
Cancellous bone	-		777	(689–800)	
Subcutaneous tissue	-		760	(701–824)	
Knee joint	-		750	(689–817)	
Plasma	-		742	(689–800)	

* T_max_ is not applicable to the group receiving continuous administration at assumed steady state. AUC_12–18 h_; area under curve from 12 h to 18 h. C_max_; peak drug concentration. AUC_tissue_/AUC_plasma_; tissue penetration. T_max_; time until C_max_.

## Data Availability

The data are available from the corresponding author upon reasonable request.
